# Effective strategies to reduce pain and anxiety in infants during routine needle-related medical procedures

**DOI:** 10.1186/s12887-026-07042-8

**Published:** 2026-05-27

**Authors:** Karin Cato, Hanna Andersson, Eva-Lotta Funkquist, Karin Enskär, Marie Golsäter

**Affiliations:** 1 Department of Women’s and Children’s Health, Uppsala University, Dag Hammarskjölds väg 14 B, 752 37, Uppsala, Sweden; 2 Child Health Services, Jönköping, Region Jönköping County Sweden; 3 CHILD Research Group, School of Health and Welfare, Jönköping University, Jönköping, Sweden; 4 Child Health Services and Futurum - Academy for Health and Care, Jönköping, Region Jönköping County Sweden

**Keywords:** Comfort Theory, Needle-related procedures, Pain management, Infants, Breastfeeding

## Abstract

Needle-related procedures are painful for infants, highlighting the need to enhance the use and understanding of non-pharmacological strategies to alleviate pain and anxiety during such procedures. This study aims to explore how needle-related routine care procedures, such as vaccinations and blood samples, are designed and described by parents and healthcare professionals to minimize pain and anxiety in newborns and infants aged 3 and 5 months, in the framework of Kolcaba’s Comfort Theory. Using a convergent mixed-method approach, which combines qualitative and quantitative data collection and analysis, the results are presented according to the three pillars of Comfort Theory: relief, ease, and transcendence. In total, 16 healthcare professionals, 40 parents, and 27 infants aged 0–5 months were included in the study. The findings describe how needle-related procedures were carried out and perceived by healthcare professionals and parents. Several key aspects were identified as important for alleviating pain, including support for parents, pain relief, preparation for parents, support and comfort for the infant, ensuring safety for the infant and experiences of well-being. The study suggests several practices to decrease pain and anxiety for infants undergoing needle-related procedures in routine care. These include supporting and preparing parents with information about the procedure and possible pain relief methods for the infant, fostering a calm environment, and ensuring the safety of the infant throughout the process.

**Trial registration** ISRCTN 12280133. Registration date: 24/04/2023.

**Supplementary Information:**

The online version contains supplementary material available at 10.1186/s12887-026-07042-8.

## Introduction

During the first months of life, infants undergo several needle-related medical procedures, such as blood sampling and vaccinations, as part of routine care. While these procedures may be medically necessary, they can still be harmful to the infant. Numerous studies have demonstrated the adverse effects of pain and anxiety associated with needle-related procedures and their impact on both short- and long-term outcomes [1–4]. Overall, ignoring the pain experienced during routine needle-related medical procedures can lead to a culture of poor pain management practices, thus failing to provide care that meets the needs of the infant [5, 6].

Several non-pharmacological strategies have been presented to reduce pain and anxiety in infants in connection with needle-related medical procedures. Breastfeeding during these procedures can be beneficial [7], as it leads to reduced pain, as indicated by based on physiological responses as decreased heart rate [8] and shorter recovery time for the crying infant after vaccination [9]. For infants who are not breastfeeding, an oral sucrose or glucose solution is recommended in connection with the procedure. Given just before the procedure, sweet solution has both pain-relieving and distraction effects [10–14]. Likewise, skin-to-skin contact has been demonstrated to be an effective method for relieving pain, primarily due to its ability to promote physiological regulation [15]. Furthermore, allowing the infant to suck on a pacifier or, for example, a parent’s finger is another strategy for reducing pain and anxiety during needle-related medical procedures [7, 16] .

One of the first painful needle-related procedures experienced in life, for most infants in Sweden, apart from the vitamin K injection given to newborns at birth, is the phenylketonuria (PKU) blood sample, followed by the three and five-month vaccination doses. For infants, their parent’s presence and collaboration with healthcare professionals (HCPs) are crucial in reducing pain and anxiety. Therefore, it is essential that HCPs are knowledgeable and sensitive to the parents’ experiences and feelings before the medical procedures [2]. Hence, HCPs must inform, prepare, and plan the procedure in collaboration with the parents to prevent unnecessary pain and anxiety for both the infant and the parent [7]. This collaborative approach is vital for understanding and implementing effective pain and anxiety management techniques during routine needle-related procedures for infants.

HCPs can support parents and their infant during the unfamiliar and stressful situation when the infant is undergoing a needle-related procedure by applying the Kolcaba’s Comfort Theory [17]. This theory is based on a holistic view of humanity, emphasizing the concepts of relief, ease, and transcendence. Relief is achieved when an unpleasant sensation is mitigated or alleviated. Ease is characterized by satisfaction, where the person feels calm and secure. The ability to rise above pain or discomfort is the state known as transcendence, which includes being able to face the inevitable pain or discomfort in a manageable way [18]. An infant’s need for relief, ease, and transcendence during routine needle-related procedure is dynamic, and the infant can thus oscillate between these needs. To reduce pain and anxiety and promote health, HCPs should adopt a holistic approach to comfort, carefully designing care and support for both the infant and parents [17–19].

Therefore, this study aims to explore how routine needle-related care procedures, such as vaccinations and blood samples, are designed and described by parents and HCPs to minimize pain and anxiety in newborns and infants aged between three and five months, within the framework of Kolcaba’s Comfort Theory.

## Method

### Design

The study was conducted using a convergent mixed-method approach, in which qualitative data (interviews) and quantitative data (study-specific observational protocol) were collected in parallel and then combined in the analysis [20]. The deductive analysis was based on Kolcaba’s [18] Comfort Theory, focusing on the concepts of relief, ease, and transcendence. This study is part of the research project: *Pain Initiative: Pediatric Procedure Intervention* ISRCTN 12,280,133.

### Setting

In Sweden, the PKU test is part of the newborn screening offered to all infants and currently includes 26 rare, treatable congenital diseases. The PKU test should be performed as soon as possible after 48 h of age, usually by a midwife or nurse at the maternity ward. If this is not feasible, it might also be taken by HCPs in a neonatal ward or lab staff. The PKU screening is conducted via venipuncture, which is the standard procedure for newborn blood sampling in Swedish maternity wards.

Child Health Services (CHS) nurses have the task of offering and vaccinating children according to the national vaccination program [21]. The combination vaccine protects against six diseases (diphtheria, tetanus, whooping cough, polio, hepatitis B, and Hib). This means that the vaccination given at three and five months of age consists of two shots, as pneumococci vaccination is not included in the combination vaccine but is given separately as an intramuscular injection [21].

### Participants

A total of 16 HCPs, 40 parents, and 27 infants were included in the present study. Healthcare professionals were invited to participate before the needle-related procedure at the maternity ward or before the infant’s visit to the CHS for an appointment.

Between January to June 2023, parents of newborns scheduled for PKU screening at the maternity ward of a University hospital in central Sweden were invited to participate in the study on days when the researcher was present.

Similarly, from December 2022 to March 2023, CHS nurses and parents in one region of southeastern Sweden were invited to participate on days when the researcher was present. Parents who attended CHS with their three or five-month-old infants for vaccinations according to the national vaccination program.

### Measuring instruments

#### Observation protocol

The observation protocol used in this study was prepared by the research group of the larger project *Pain Initiative: Pediatric Procedure Intervention* (ISRCTN 12280133). This study is part of a larger research project that includes children of various ages, which explains the use of child-related terminology in the broader context. However, it is important to emphasize that the current study being reported here exclusively includes infants. The protocol was intended to be used to observe and document HCPs’ strategies for reducing pain and anxiety in infants during needle-related procedures. The protocol was structured under the following headings: “The HCP creates security by…”, “The HCP provides information and preparations by…”, “The HCP diverts and distracts the child by…”, and “The HCP creates safety for parents by…”. The study-specific protocol was developed from literature, inspired by previous instrument, including pain- and anxiety preventing strategies related to: Security, Participation, Information, Distraction, Relaxation, and Avoiding forcibly hold.

#### Semi-structured interviews

The interviews with parents and HCPs followed a semi-structured interview guide based on two overall questions. The interviews with parents were based on the questions: What strategies did the HCP use during the procedure to reduce anxiety and pain in your child? What strategies did you use as a parent?

In the interviews with HCPs, the following questions were asked: What strategies did you use as an HCP to reduce the child’s anxiety and pain during the procedure? What strategies did the child’s parents use? The interview guide is to be found as Supplementary material.

#### Pain in children: Face, Legs, Activity, Cry, Consolability-scale (FLACC)

The FLACC scale consists of five observation areas, which are graded and then scored from zero to ten. A score of zero indicates no pain, while ten represents the highest level of pain; a score above three indicates the presence of pain. The different observation areas are translated into face, legs, activity, crying, and consolability [22]. The FLACC scale is validated for estimating pain in infants undergoing procedure-related pain [23].

#### Anxiety in parents: Visual Analog Scale – Anxiety (VAS-A)

The VAS-A is a ten-centimeter horizontal line where individuals mark their perceived level of anxiety regarding a specific situation, for example, a painful needle-related procedure. The left end of the line is described as “No anxiety at all,” and the right end is labeled “worst possible anxiety.” The line can be divided into ten parts, and a mark above four may indicate worry or anxiety about the situation. It is a simple method that is well-established in healthcare and has been validated for children (> four years) as well as for adults [24, 25].

### Data collection

Quantitative data were collected through the observation of routine needle-related procedures (PKU testing and vaccinations) at the maternity ward and during health visits at CHS. The study-specific observation protocol and two scales, Visual Analog Scale—Anxiety (VAS—A) and the Face, Legs, Activity, Cry, Consolability-scale (FLACC) were used to gather this data. The data collection followed three steps; (a) before the procedure, (b) during the procedure, and (c) after the procedure. Before the needle-related procedure (a), the included HCPs answered background questions regarding their age, years in the profession, and experience working with children (years). Likewise, the included parents answered background questions regarding themselves and their infant. FLACC was observed in the infant, and the parents filled in VAS-A.

**Fig. 1:**
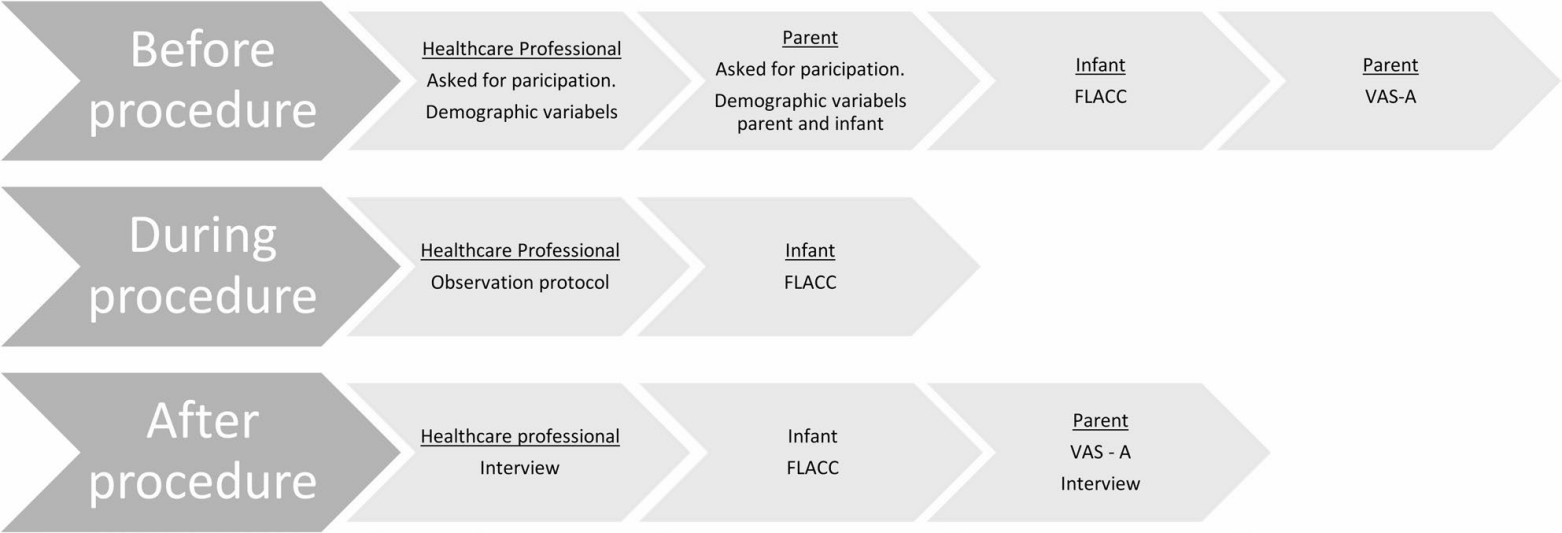
Overview of the data collection procedure

The observer was present throughout the needle-related procedure (b) without completing any tasks. The procedure was observed based on the observation protocol, and the infant’s pain was assessed based on the FLACC scale. The observer positioned herself in the room so that the HCP, the infant, and the parents could be observed. The observation protocol was completed continuously as the HCP prepared the infant and parent until the procedure was completed. Field notes were taken during the observation and included to support the subsequent interviews. After the procedure (c), the HCPs were interviewed. FLACC was observed in the infant, and the parents were interviewed and filled in VAS-A once more. Figure 1 shows an overview of the data collection procedure.

### Data analysis

Since the study utilized a convergent design based on mixed methods, parallel analysis was performed. This meant that quantitative and qualitative data were first analyzed separately and then compared and interpreted [20]. Below is a presentation of the steps taken in the analysis process, from being analyzed separately to being compared and interpreted.

### Quantitative data

The observation protocol was compiled and presented using descriptive statistics, highlighting the strategies used and their frequency. The Vas-A and the FLACC-scale were analyzed using descriptive statistics to determine the degree of pain and anxiety.

### Qualitative data

The qualitative data underwent deductive content analysis based on the model by Elo and Kyngäs [26]. An unlimited matrix was used, which means that the matrix was created based on the chosen theory, which in this study was Kolcaba’s Comfort Theory [18]. The analysis followed a deductive process, where categories were created within the framework of the theory [26]. The analysis then went through the three steps as described by Elo and Kyngäs [26]: the preparation phase, the organization phase, and the reporting phase. In the preparation phase, all interviews were read through by several members of the research group to gain a deeper understanding of the text. The interviews were then discussed and compared within the research group.

The organization phase began with the establishment of a matrix based on the three states described by Kolcaba [18]: relief, ease, and transcendence.

An analysis matrix was established for the HCPs’ interviews and for the parents’ interviews. Text deemed relevant to the purpose was selected and placed into the appropriate sections of the matrices under the corresponding headings. The selected text was then condensed. Thereafter, the two matrices were compared, and similarities and differences were identified. These were found to complement each other, which led to the text in the two matrices being combined into one matrix. The last step in the qualitative analysis, the reporting phase, involved grouping the condensed sentences that were judged to belong together. After the grouping, subcategories were created and compiled into the category matrix. The category matrix was then analyzed, and the sentence units, condensation, and subcategory content were tested to ensure alignment with the categories.

### Interpretation of analysis

After analyzing both qualitative and quantitative data, the results were compared. Similarities and differences were viewed as complementary. Compiling the findings into the category matrix created during the qualitative analysis enriched the results, as both quantitative and qualitative data were described together. This interpretation of both types of data aligns with the models described in a convergent design for mixed methods research [20].

This study was conducted in accordance with the ethical standards set by the Swedish Ethical Review Authority, registration number 2022-00944-01, and the 1964 Helsinki Declaration, along with its subsequent amendments. Before the observations and the interviews, participants received written and oral information about the study procedures and gave their written consent to participate. In the case of minors, parental informed consent was obtained for participation.

## Results

In total, 27 infants, 40 parents and 16 HCPs were included in the study. Demographic data for the participants are presented in Table 1. Pain in the infants was measured using the FLACC scale, which indicated that infants mainly exhibited signs of pain during the needle-related procedure. Newborns had higher FLACC scores before the procedure compared to older infants. Parents did not report high anxiety levels on the VAS-A scale. Data for FLACC and VAS-A are presented in Table 2.

**Table 1:** Demographic variables of the participants

Children	*N* (%)
Number	27 (100)
Gender	
* Boys* * Girls*	12 (44) 15 (56)
Age	
* Newborn* * Three months* * Five months*	15 (56) 8 (30) 4 (14)

Parents	
Number	40 (100)
Gender	
* Female* * Male*	24 (60) 16 (40)
Age (years)	
* 20–30* * 31–40* * 41–50*	16 (40) 21 (53) 3 (7)
First-time parents	22 (55)

Healthcare professionals	
Number	16 (100)
Gender	
* Female* *Male*	16 (100) 0 (0)
Age (years)	
* 25–44* * 45–55* * 56–65*	10 (63) 3 (18.5) 3 (18.5)
Professional title	
* CHS-nurse* * Midwife* * Nurse*	5 (30) 9 (56) 2 (4)
Length of professional experience (years)	
< 2 2–5 6–20 > 20	2 (12.5) 5 (31) 6 (38) 3 (18.5)

**Table 2:** FLACC and VAS-A

Children	
FLACC mean (range)	
* 5 min before the needle procedure* * During the needle procedure* * 5 min after the needle procedure*	0.9 (0–8) 3.6 (0–9) 0.04 (0–1)

Parents	
VAS-A mean (range)	
* before the needle procedure* * after the needle procedure*	0.8 (0–7) 0.5 (0–5)

The results are presented in two parts. First, figures illustrate the strategies observed in the postnatal ward and at the CHS according to the categories of Kolcaba’s Comfort Theory. Second, the combined findings from observations and interviews are presented, organized by categories and sub-categories in alignment with the same framework.

### To create relief during the needle procedure

**Fig. 2:**
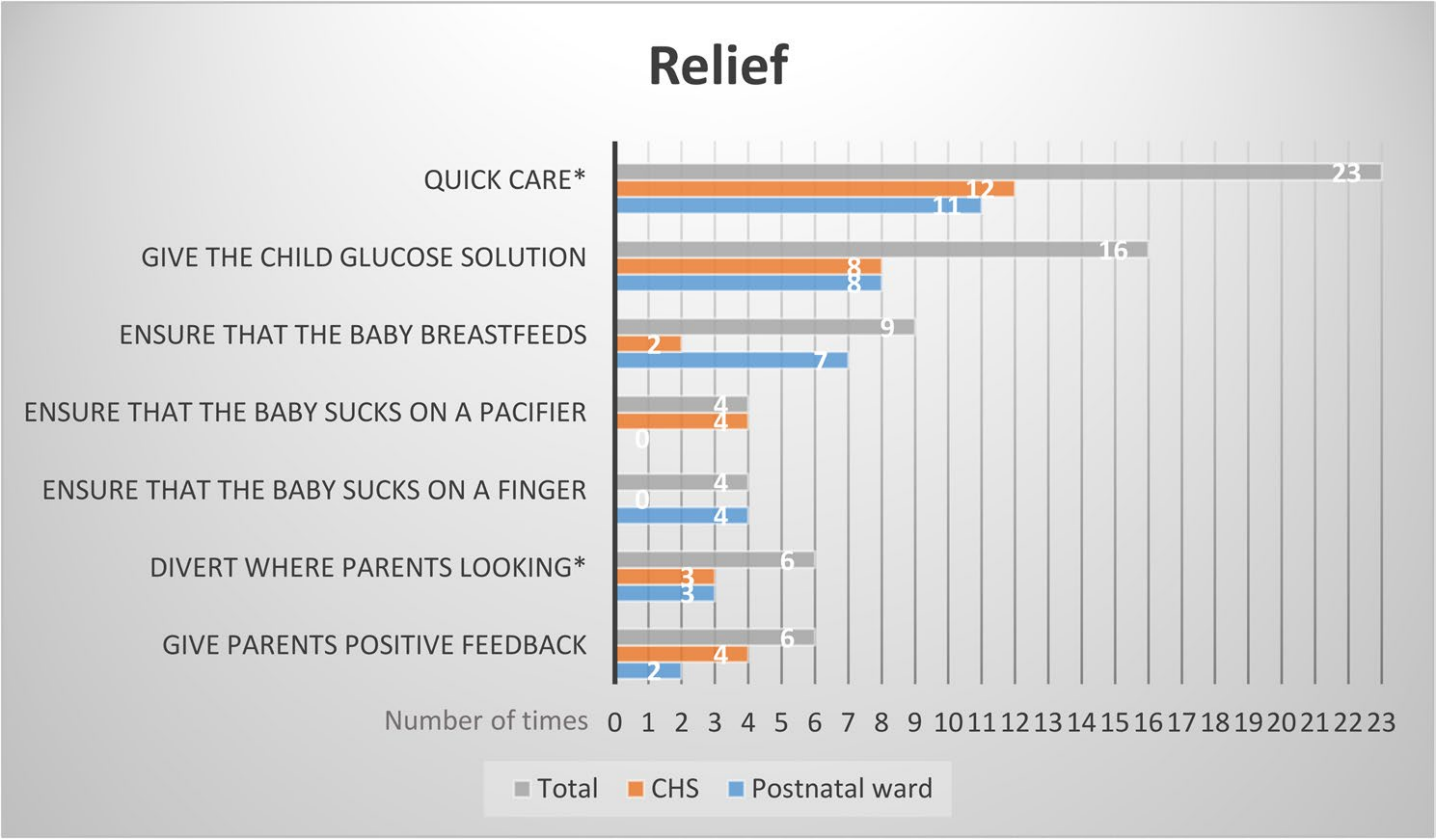
Number of times (*N* = 68) different relieving strategies were observed at both the postnatal ward and in the CHS. * *Quick care*; the HCP didn’t prolong the procedure unnecessarily, *Divert where parents looking;* can be a strategy to relief the parents’ anxiety, which possibly can affect the infant

To create relief, the HCPs emphasized the importance of considering both the infant and the parents as a unit throughout the entire needle procedure. Observed relieving strategies are presented in Fig. 2. The parents expressed their wish to protect their infant from pain and portrayed how the HCPs supported them in ensuring their infant’s comfort during the needle-related procedure. The category *To create relief during the needle procedure* is divided into two sub-categories: *Supporting the parents* and *Relieving the pain induced by the needle procedure*.

### Supporting the parents

Supporting the parents was considered a significant aspect of alleviating pain and anxiety in the infant. The HCPs gave instructions on how the infant could be placed during the procedure, encouraging the parents to hold their infant in their arms. This was observed in almost all needle procedures, except for a few cases where the infant slept throughout the procedure in a cot. If the infant was breastfed, the HCPs encouraged breastfeeding while carrying out the needle procedure, which was also observed during various procedures. Parents described how they got involved in the procedure with support from the HCPs:

*No*,* it’s just to check with the mother how she wants it to be done–if you should*,* if you want to breastfeed or if you want it to go fast*,* or if you should take one [vaccination] at a time and comfort in between*,* or yes*,* you check with the mother about what you want. How to do it. (Parent*,* interview 3)*

Simultaneously, the HCPs described how they engaged the parents to be actively involved:

*I think it works best when they are breastfeeding*,* but I always want to have sugar ready*,* so you can give it if needed […] If necessary*,* I want the father or mother to give a little sugar right away*,* so that the baby stays calm. (Midwife*,* interview 2)*

Another supportive strategy involved providing positive feedback about the importance of parental presence for the infant. Furthermore, asking parents how they felt about the needle procedure beforehand, or addressing any fear of needles, was a way of providing support. HCPs suggested that the parent/s look away in order to divert them from the actual puncture of the skin or provided strategies to console the infant.

*If you know which parents you have in front of you–those who are nervous and scared and such– I try to focus on the fact that it shouldn’t*,* well*,* I try to calm them down. And for those who you don’t know*,* well then*,* you have to explain how it will go*,* that the baby will scream and that you can hold them afterward and such. And that it hurts*,* it stings*,* but it passes quickly. (CHS nurse*,* interview 12)*

*Now*,* our girl had already eaten a lot and was really tired. She was just sleeping. There was no point in breastfeeding. So*,* it was smart to be able to soothe her with a finger if she had gotten upset. (Parent*,* interview 11)*

To alleviate a worried parent, HCPs often took extra measures for the infant, such as massaging the infant’s thighs before the vaccination or talking directly to the infant before, during, and/or after the blood sample. The experience of the needle-related procedure differed between the parents. Some expressed anxiety and described the importance of receiving support from the HCP to manage the situation. Conversely, parents who did not express any worry stated that they felt a sense of security with the HCP, which provided sufficient support.

### Relieving the pain induced by the needle procedure

Both HCPs at the CHS and attending parents described the challenge of providing pain relief to infants during the vaccination. At the maternity ward, however, this concern was not at the same level among HCPs and parents. Several non-pharmacological strategies for pain relief were identified by parents and HCPs during needle procedures. All infants at the maternity ward and many at the CHS were breastfed before, during, and/or after the procedure, or given formula. Both parents and HCPs described this strategy as soothing, diverting, and satisfying for the infant, making it an effective strategy to minimize pain and anxiety. Moreover, at the maternity ward, the HCPs encouraged parents to keep their infant skin-to-skin to provide pain relief, although this strategy was not mentioned by parents. All participating HCPs and parents interpreted the infant’s relief by the absence of crying, or if the crying stopped quickly after the needle procedure. Furthermore, it was described that performing the needle procedure while the mother was breastfeeding could be more challenging for the HCPs, as it was sometimes difficult to reach the insertion area. However, the soothing effect of breastfeeding was considered more important than an adequate working position. Additionally, some newborns and all three-month old infants were given glucose/rotavirus vaccine, respectively, orally before the needle procedure. This was another strategy used by the HCPs, as the sweet solutions of glucose and the rotavirus vaccine helped to divert the infant’s attention and reduce discomfort.

*No*,* but partly it’s about trying to establish some contact with the child and then giving the rota vaccine first. It still provides like a bit of pain relief*,* and then we try to be as quick as possible. (CHS nurse*,* interview 14).*

*I usually make sure that they are always in the parent’s arms and then offer sugar. Alternatively*,* that they are breastfeeding. (Midwife*,* interview 14)*

All infants receiving vaccinations at the CHS were given two injections simultaneously, one in each thigh, to ensure a fast procedure. Both HCPs and parents at the maternity ward mentioned that a fast procedure was crucial to alleviate pain and anxiety.

*She (the CHS nurse) is so fast; she does it so fast; she prepares everything and so on*,* it goes really fast and then you feel calmer too. If it had taken a long time and that it had been long*,* it would have been much harder and worse. (Parent*,* interview 11)*

At the maternity ward, all HCPs employed a specific strategy to expedite the needle procedure. They used a medical glove filled with warm water on the infant’s hand before the needle procedure, ensuring the veins were more visible and increasing the likelihood of a quick and easy blood draw. This was also noted as a pain relief strategy by the parents, although it was not certain that they understood the mechanism behind it.

*She (the midwife) used the glove to warm and kind of gently press on the hand. I guess it’s some kind of pain relief? (Parent*,* interview 7).*

Other ways to ease pain included diversion, especially at the CHS. This involved talking and/or singing to the infant, playing music, using a rattle, and/or blowing soap bubbles. If siblings were present, they could be engaged to help divert the infant’s attention. Using a pacifier before, during, and after the vaccination was also described by parents and HCPs as providing pain relief. None of these specific strategies were used at the maternity ward; instead, breastfeeding and skin-to-skin care were the most common strategies to relieve pain during the needle procedure.

### To create ease during the needle procedure

**Fig. 3:**
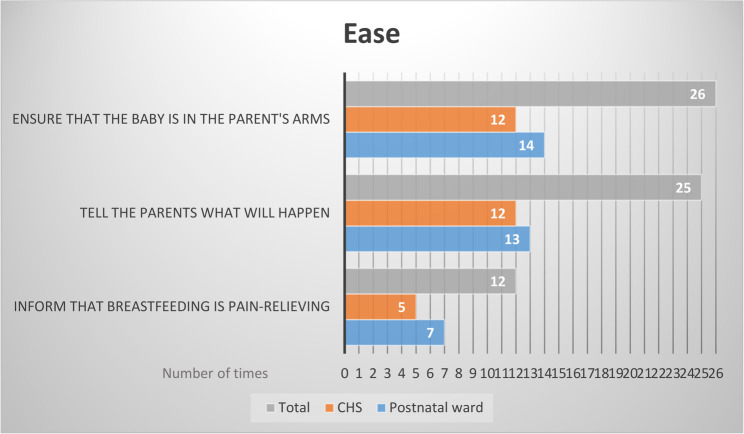
Number of times (*N* = 63) easing strategies were observed at both the postnatal ward and CHS

Creating a sense of ease where the infant was calm was depicted by both HCPs and parents as an important part in decreasing the infant’s pain and anxiety. By assuring that the parents were calm, the infant also remained calm during the procedure. The strategies to create ease during the needle procedure are compiled into two sub-categories, namely *Preparation for the parents* and *Support and comfort for the child*. The observed strategies aimed at creating ease are presented in Fig. 3.

### Preparations for the parents

Providing information about the needle procedure was described by both HCPs and parents as essential for creating a sense of ease and calm for the infant. This strategy was also commonly observed. Well-informed parents felt more secure and at ease, enabling them to better support their infant during the procedure.

*No*,* but it was to prepare the mother for what will happen and tell her exactly what to do; that is important. (CHS nurse*,* interview 22).*

A parent at the maternity ward shared:

Yes, *it doesn’t feel great that they are going to poke your child*,* but you do it anyway because it is important for the child’s future. If they are sick or something*,* it’s important to know. (Parent*,* interview 14).*

The parents emphasized the importance of receiving information about the vaccination during an earlier appointment at the CHS, in order for them to prepare and plan the visit for both themselves and their child. Parents with more than one child highlighted the need to receive repeated information about the vaccination, since they had forgotten the details between the births of their children. They also valued guidance on how to support their infant during the procedure. The HCPs described how they tailored the information to meet the individual needs of parents. They described how the needle procedure would be carried out and explained to the parents when and how to comfort their infant during the procedure. Parents described how receiving information about the potential side effects of the vaccination could help ease their anxiety:

*Yes*,* but I think so*,* I have learned that if she gets a fever*,* it’s because of it… If she’s in pain*,* it is because of that… And if we notice something out of the ordinary when at home*,* I don’t have to worry because then I know what it could be due to. (Parent*,* interview 17)*

The structure of the health visit was another important issue when the HCPs described how they helped parents feel at ease during the vaccination. HCPs typically started the visit with routine checkups and terminated with the needle procedure, so that the parent could focus on comforting their infant afterward if needed. Other preparations could be letting the parents choose which of them, if both were present, would hold the infant during the needle procedure. Parents and HCPs at the CHS as well as the maternity ward noted that the parent who felt least anxious usually held the infant. Similarly, parents at both places described how they looked away during the needle puncture in order to avoid anxiety from seeing the needle. Having both parents present, or a support person for a single parent, was also described as supportive for anxious parents. The HCPs acknowledged that if the room was crowded with supportive persons or siblings, it could be harder to create a calming environment. However, involving each person present in different tasks to support the infant could make their presence helpful during the needle procedure. If no support person was available, or if the present parent was too anxious to hold the infant, HCPs could arrange for another HCP to assist. Parents expressed that understanding the long-term benefits of PKU screening or vaccination for their infant’s health motivated them to proceed with the needle procedure even though they were anxious.

### Support and consolation for the child

Parents were well aware that their feelings could affect their infant and had, therefore, developed strategies to manage their own anxiety. Both parents and HCPs emphasized the importance of ensuring the infant was calm and satisfied before the needle procedure. They described this, for example, by making sure that the infant had eaten before the needle procedure, with breastfeeding particularly noted as a comforting activity for both the child and mother. The HCPs also described their own preparations as crucial strategies to support and console the infant.

*That I should be prepared*,* reviewing which vaccines will I be giving*,* always going through: is it the right vaccine*,* is it the right child*,* everything around that we have as a strategy or guidelines. (CHS nurse*,* interview 4)*

*So*,* first I go in and put on heat and then I ask the parent to breastfeed the child*,* so that the infant is full when it is time for the procedure. (Midwife*,* interview 12).*

Both parents and HCPs believed that the infant would feel safer in the parent’s arms, where the parent could provide comfort throughout the needle procedure. This sentiment was not only described, but also observed by the researcher.

*She got to be close to us; that’s what’s best for her. Safest. (Parent*,* interview 1)*

*I think that from the staff’s point of view*,* it’s best to do the test on the examination table because you have the right height*,* good lighting and such. But it’s better for the child and the parents if you go to them and do it*,* so you do that. (Midwife*,* interview 15)*

While in their arms, parents could hold their infant, nurse, offer a pacifier or a finger (possibly with a drop of glucose), talk calmly, or hold hands. Furthermore, the parents noted the importance of staying calm themselves, as their calmness transferred to their infant. The HCPs emphasized the importance of a satisfied infant before the needle procedure and therefore waited until both the infant and parent/s were ready to proceed. After the vaccinations, infants at the CHS were typically crying and upset. Conversely, newborns at the postnatal ward often cried more intensely before the needle procedure, mainly because they were awakened to be placed at the breast. However, they calmed down afterward, often as soon as nursing began. The parents and the nurses at the CHS described needing to comfort the infants afterwards, while the parents in the postnatal ward felt relieved that the needle procedure went so smoothly. A variety of strategies to comfort the child were described by both HCPs and parents at the CHS. The main strategy involved the parent holding the infant in their arms. They consoled their infants by holding them close, caressing them, offering a pacifier, talking or singing to them, rocking them, distracting them by looking at different things in the room, and/or offering something to eat, or breastfeeding.

### Achieving manageability during the needle procedure

**Fig. 4:**
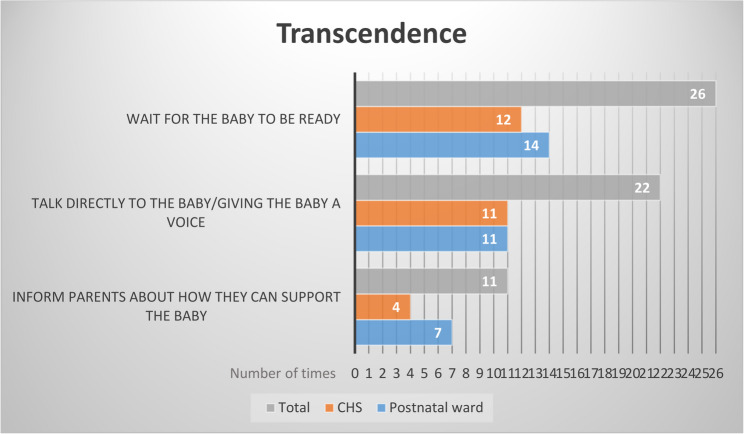
Number of times (*N* = 59) observations of transcendence took place at both the postnatal ward and the CHS

Several strategies to achieve manageability during the needle procedure were observed by the researcher. These strategies are presented in Fig. 4.

When the infant was calm after the procedure, both the HCPs and the parents described the experience as manageable and even joyful. The concept of finding manageability during the needle procedure and how it was experienced is explored through the sub-categories of *Safety for the infant* and *Experiences of well-being*.

### Safety for the infant

Creating a safe environment for the infant was recognized by both HCPs and parents as essential to reducing pain and anxiety during the needle procedure. To ease anxiety, it was considered important for the infant to feel satisfied and secure.

*I pointed out that if the mother could breastfeed the girl first*,* because it’s partly*,* because it is pain-relieving and if she is full and satisfied*,* she will feel more secure and calm … (CHS nurse*,* interview 6)*

If the infant was hungry or expected to become hungry, both nurses and midwives noted that this could affect the needle procedure negatively. Nurses at the CHS, but not midwives at the maternity ward, also stated that if the infant was tired, it could be troublesome. The HCPs explained that it was challenging to create a safe environment if the parents were anxious and/or nervous. To calm their infant, the parents tried to focus on things other than the needle procedure. Furthermore, the HCPs described that they focused a great deal on creating calm parents. If the parents were calm, the likelihood of the infant staying calm or being soothed easily after the needle procedure increased. The HCPs further described that a secure attachment between the infant and the parent was crucial for keeping the infant calm during the procedure. A safe attachment also helped the infant to recover smoothly, as described by the parents:

*No*,* it’s more that he is comforting himself; it’s clear that he is more at ease with mom. He may notice that I’m comforting him as I usually do. (Parent*,* interview 15)*

Similarly, the HCPs described that a prompt response from the parents in comforting their infant was an effective strategy to calm the infant after the needle procedure, if needed.

*Yes*,* if we think about this case*,* I think they comforted*,* that is*,* when the vaccination was finished*,* they turned the child quite quickly towards them and comforted and got up and walked around with the child*,* talking calmly. (CHS nurse*,* interview 2)*

The HCPs provided support and guidance to the parents on how to comfort and distract their infant. The nurses at the CHS noted that talking to the infant was a diversion strategy. This could involve explaining that “it will sting” or even pretending to be the child and giving the infant a voice. The midwives at the maternity ward also used this strategy to create a sense of safety for both the infant and the parents. Parents, both at the maternity ward and at the CHS, mentioned that their previous experiences with older children’s needle procedures, such as vaccinations or neonatal intensive care, had equipped them with strategies that they could use to create a safe environment during the current needle procedure.

### Experiences of well-being

The well-being of the infant was described by parents as their child being happy, calm, or asleep after the needle procedure. This sense of well-being brought feelings of joy to both the parents and the HCPs.

*No*,* now after the vaccination*,* they’re sleeping and calm*,* and so am I. (Twin parent*,* interview 7)*

Both HCPs and parents at the postnatal ward described that a shorter duration of screaming-time was often linked to the infant being comforted through breastfeeding. The duration of the infant’s crying after the needle procedure, until comforted and calm, was regarded as an indicator, by both the HCPs and parents, of the procedure’s success. A brief or absent crying period was seen as a positive reflection of how well the whole procedure went.

## Discussion

This study describes strategies used by HCPs to minimize pain and anxiety during routine needle procedures for newborns and infants at three and five months of age. Support for the parents was an important part in reducing the child’s pain and anxiety. Through information, individualized support, and fostering a secure relationship between the HCPs, the child, and the parents, the needle procedure could be performed in a manageable way. Parents shared how they prepared themselves before the needle procedure and how the information and support from HCPs were helpful. Furthermore, the results showed the importance of ensuring the child was content and calm during the procedure. Several strategies were implemented while the child was held skin-to-skin or in the parents’ arms, before, during, and after the needle procedure.

The main themes in this study follow the Comfort Theory of *Relief*, *Ease*, and *Transcendence*. *Relief* was achieved through the support provided by HCPs to the parents and by alleviating the pain experienced by the children during the needle procedure. *Ease* was facilitated by preparing the parents beforehand and offering support and comfort to the child during the procedure. Transcendence was realized when HCPs prioritized the child’s safety and when the needle procedure could lead to experiences of positive well-being for both the child-parent dyads and the HCPs.

### Relief

Support for the parents and strategies to alleviate pain were the two key aspects of relief observed in this study. Newborns and infants aged three to five months are totally dependent on their parents, making the support provided by HCPs to the parents an important factor in achieving relief for the child. Including parents in the process is one of the most effective ways to alleviate pain during needle-related procedures in children [27]. Similarly, in the interviews, parents described how the support from HCPs helped them console their child during the procedure. They described how they felt involved in their child’s care; moreover, the relationship and collaboration between parents and HCPs were considered important for both the HCPs and the parents in creating relief for the child.

There is a plethora of evidenced-based non-pharmacological strategies that may be used during needle procedures in infants and children, such as breastfeeding, skin-to-skin care, and the use of glucose solutions [28]. In the present study, we included the use of glucose solution as a non-pharmacological intervention, in line with previous research. It was administered as a complementary measure when the infant was not breastfeeding. However, its classification as a non-pharmacological could be subject to debate since analgesic effects of oral sweet solutions are considered to be endogenous opioid mediated. To get relief for the child, the results of this study confirm the use of many non-pharmacological strategies in reducing pain associated with needle procedures. The non-pharmacological strategies that were identified are similar to those found in other studies, such as breastfeeding, closeness to the parents, i.e., skin-to-skin or being held in a parent’s arms, and oral glucose [29]. Breastfeeding has been shown in some studies to be more effective compared to glucose and distraction techniques [30], while the systematic review by Shah et al. concluded that breastfeeding and breastmilk were no more effective than oral sweet solutions [8]. However, breastfeeding was not extensively used as a strategy by the HCPs and parents in this study. While it was mentioned in the interviews with the HCPs that breastfeeding is one of the best strategies for alleviating pain in infants, it was mentioned that practical challenges, such as ergonomic working positions or the infant’s lack of an apparent need for pain relief at the time, influenced its usage. Breastfeeding is widely considered as the best non-pharmacological strategy for pain relief, highlighting the importance of raising awareness among HCPs about this strategy when performing needle-related procedures on infants and children [31]. Furthermore, performing the needle procedure as quickly as possible was another relieving strategy used by HCPs, which was noticed and appreciated by the parents. However, there was some concern that some HCPs at the CHS gave two injections simultaneously, one in each thigh during the vaccination. One could speculate that giving the vaccines one after another might reduce prolonged pain, simultaneous injections could be perceived as more frightening and painful due to the pain occurring in different places at the same time. A review on pain reduction during pediatric immunizations suggests that parents generally prefer simultaneous injections if possible [32], though it is probably based on the HCPs’ recommendation. Furthermore, administering simultaneous injections makes it difficult to breastfeed at the same time, which means that the chance of using breastfeeding as a pain-relieving method is reduced.

### Ease

Parental information about the specific pain-related procedures has been suggested to empower parents in alleviating their children’s pain during medical procedures [33], as was evident in the present study. The results depict how both the HCPs and parents strived to create ease for the sake of the child. Information about the PKU test or vaccination was provided and later described as important for achieving calmness and ease during the needle procedure. Additionally, the structure of the health visit could be of importance in fostering a smoother procedure. At the CHS, it was considered standard practice to end the visit with the vaccination, hence preventing eventual problems in performing other examinations included in the visit caused by an upset child. However, the FLACC scores differed between newborns in the maternity ward and infants at the CHS. The newborns in the maternity ward exhibited higher scores before the needle procedure. This was probably because newborns who were placed at the breast before the procedure showed signs of fussiness, such as whining and/or crying, before starting to suckle. These higher FLACC scores were most probably not signs of pain but rather hunger or a need for closeness.

Furthermore, to support and comfort the child, most infants in the present study were either in skin-to-skin-contact with a parent or held in the parent’s arms. This allowed the parents to console their child during the procedure, for example, breastfeeding or by distracting the child in different ways. This is in line with previous studies, concluding that parental presence and involvement may decrease pain and anxiety in children undergoing medical procedures [34].

### Transcendence

The need to feel trust in the HCP and the motivation to cope with pain and discomfort are included in the psychological need for comfort. This need forms an excellent foundation for the infant and parents to achieve transcendence. The socio-cultural need encompasses the factors surrounding the infant and parents, including the HCP’s ability to create a secure, calm, and positive atmosphere, as well as the influence of social networks and cultural traditions. Involving parents and guiding them in how to create a sense of security highlights their significant role — security is constituted by the presence of a parent, as also described by Sahlberg et al. [35]. Both HCPs and parents described the needle procedure as manageable when the infant remained calm during the procedure or could be readily soothed by its parents afterwards. Bice et al. [36] has described that parents may perceive nurses’ ability to engage in small talk during needle-related procedures as a means of providing psychological and social comfort. Similarly, our results show that providing parents with information could help mitigate anxiety, and including the infant by speaking directly to them enhanced a sense of transcendence. To foster a health-promoting environment, HCPs should consider the infant’s senses as a starting point by, for example, reducing noise, avoiding bright lights, and eliminating unpleasant smells [17, 18].

### Methodological considerations

The mixed-methods design was deemed the most suitable since the aim of this study was to explore strategies to minimize pain and anxiety during needle procedures in newborns and infants three to five months of age. Quantitative and qualitative data complement each other, with the strengths of qualitative data compensating for the weaknesses of quantitative data, and vice versa [20]. This method also reflects the work of the HCPs, who collect measurable data, such as weight or length, together with data through conversations with parents to gather information about the child. It should be noted that only four of the participating infants were five months old, which may have influenced the overall result for this age group. Accordingly, the strategies suggested based on the findings of this study are applicable primarily to infants up to five months. For older children, strategies must be adapted in accordance with their developmental stage and level of maturity. In Swedish maternity wards, both parents are encouraged to stay throughout their total hospital stay, and are invited to participate in the CHS. This explains why the number of parents included in the present study exceeds the number of infants. None of the parents who were invited declined participation. However, five HCPs chose not to participate, and consequently, no observations were conducted involving them or their patients.

Establishing credibility and confirmability is important when conducting qualitative studies. Credibility was increased by the research team being a collaboration between specialist nurses in pediatric care and midwives. This collaboration yielded different opinions in designing the observation and question guides, as well as during the analysis process. Confirmability of the study was enhanced through the research groups’ awareness of their pre-understandings of the subject.

Nevertheless, the HCPs participating in this study may be more confident in their approach toward parents and children, especially when performing needle-related procedures, knowing they were being observed by researchers with healthcare education. Also, it cannot be ruled out that the presence of an observer in the room, or the fact that parents were asked questions while still in hospital or at the CHS, may have influenced their behavior.

## Conclusions

HCPs are aware of several evidence-based strategies to prevent, manage, and alleviate pain and anxiety in infants during needle-related procedures. However, these strategies are not fully used in healthcare, leading to children suffering unnecessarily. In the present study, the HCPs used a plethora of different strategies, which were divided into the three pillars of comfort theory as described by Kolcaba [18].

Based on the results of the present study, the following practices are recommended to decrease pain and anxiety for infants undergoing needle-related procedures in routine care, such as the PKU-test and vaccinations: support and prepare parents by providing comprehensive information about the procedure and possible ways to relieve pain for their child. With adequate information about successful strategies to decrease pain and anxiety, parents are prepared to handle future procedures in healthcare. Furthermore, enhance a calm environment and protect the child’s safety. It is crucial that painful procedures occurring early in children’s lives are conducted according to the highest standards, as these situations are likely to set a precedent for how parents support their child in the future. If the child is breastfed during the procedure, there is a higher likelihood that parents will continue to use this pain relief method in future procedures.

## Supplementary Information

Supplementary Material 1.

## Data Availability

The data used during the current study are available from the corresponding author on reasonable request.

## Abbreviations

CHS: Child Health Services
FLACC: Face, Legs, Activity, Cry, Consolability-scale
HCP: Health Care Professionals
PKU: Phenylketonuria
VAS-A: Visual Analog Scale
