# Supplementary material for: Effective strategies to reduce pain and anxiety in infants during routine needle-related medical procedures
Source: BMC Pediatr. 2026 May 27;26:498. doi: 10.1186/s12887-026-07042-8 (PMC13217710; doi:10.1186/s12887-026-07042-8)
Supplement: Supplementary file 1 — Supplementary Material 1. [file 12887_2026_7042_MOESM1_ESM.docx]

**Supplementary material; Semi-structured interview guide**

**1. Interview with Healthcare Personnel Regarding:**

**A. Strategies Used by Healthcare Personnel During the Procedure**

- What strategies did you, as a healthcare professional, apply during the procedure?
  - Were they effective from a practical/technical perspective?
  - Were they appropriate for the infant’s age, developmental level, and interests?
  - Did they help reduce the infant’s anxiety, fear, or pain?
  - In retrospect, do you think alternative strategies might have been more appropriate?

**B. Strategies Used by the Infant’s Parent(s)/Guardian(s)**

- What strategies did the infant’s parent(s) or guardian(s) use during the procedure?
  - Were they effective from a practical/technical perspective?
  - Were they appropriate for the infant’s age, developmental level, and interests?
  - Did they help reduce the infant’s anxiety, fear, or pain?
  - In retrospect, do you think alternative strategies might have been more appropriate?

**2. Interview with Parent(s)/Guardian(s) Regarding:**

### A. Strategies Used by Healthcare Personnel During the Procedure

- What strategies did the healthcare personnel use during the procedure?
  - Were they effective from a practical/technical perspective?
  - Were they appropriate for your infant’s age, developmental level, and interests?
  - Did they help reduce your infant’s anxiety, fear, or pain?
  - In your opinion, could any alternative strategies have been more appropriate?

### B. Strategies Used by You as a Parent/Guardian

- What strategies did you use during the procedure to support your child?
  - Were they effective from a practical/technical perspective?
  - Were they appropriate for your child’s age, developmental level, and interests?
  - Did they help reduce your child’s anxiety, fear, or pain?
  - In retrospect, do you think any other strategies might have been more appropriate?
